# Role of Germinated–Extruded Desi Chickpea Supplementation on Antioxidant and Antidiabetic Compounds In Vitro Bioaccessibility in Functional Blue Corn Tortillas

**DOI:** 10.3390/foods15101798

**Published:** 2026-05-19

**Authors:** Evelia M. Milán-Noris, Victor M. Hernández-Castro, Marilena Antunes-Ricardo, Alvaro Montoya-Rodríguez, Eslim S. Sandoval-Sicairos, Jorge Milán-Carillo, Cuauhtémoc Reyes-Moreno, Ada K. Milán-Noris

**Affiliations:** 1 Laboratorio de Nutracéuticos (#18), Facultad de Ciencias Químico Biológicas, Universidad Autónoma de Sinaloa, Calz de las Américas Nte 2771, Cd Universitaria, Burócrata, Culiacán 80030, Sinaloa, Mexico; evemilan@uas.edu.mx (E.M.M.-N.); alvaromr@uas.edu.mx (A.M.-R.); sugeysandoval@uas.edu.mx (E.S.S.-S.); jmilanc@uas.edu.mx (J.M.-C.); creyes@uas.edu.mx (C.R.-M.); 2 Posgrado en Ciencias de la Nutrición, Facultad de Ciencias de la Nutrición y Gastronomía, Universidad Autónoma de Sinaloa, Av. Cedros, Los Fresnos, Culiacán 80019, Sinaloa, Mexico; 3 Posgrado en Ciencia y Tecnología de Alimentos, Facultad de Ciencias Químico Biológicas, Universidad Autónoma de Sinaloa, Calz de las Américas Nte 2771, Cd Universitaria, Burócrata, Culiacán 80030, Sinaloa, Mexico; 4 Tecnologico de Monterrey, Escuela de Ingeniería y Ciencias, Av. Eugenio Garza Sada 2501 Sur, Tecnologico, Monterrey 64849, Nuevo Leon, Mexico; marilena.antunes@tec.mx; 5 Programa de Posgrado integral en Biotecnología, Facultad de Ciencias Químico Biológicas, Universidad Autónoma de Sinaloa, Calz de las Américas Nte 2771, Cd Universitaria, Burócrata, Culiacán 80030, Sinaloa, Mexico

**Keywords:** tortilla, chickpea, germination, extrusion, bioaccessibility, antioxidant

## Abstract

Corn tortillas are consumed daily in Mexico. Alkaline extrusion is an alternative process that generates nixtamalized tortillas and preserves more bioactive compounds. Chickpea germination-extrusion may enhance the bioactive compound content. The aim was to characterize the physicochemical and antioxidant/antidiabetic properties of functional tortillas of alkaline-extruded blue corn (TC) with germinated (TG) or germinated-extruded (TGE) desi-chickpea. Likewise, the effect of simulated gastrointestinal digestion (SGD) on the bioaccessibility of bioactive compounds (phenolics, soluble protein, peptides, anthocyanins, and isoflavones) was estimated. Antioxidant capacity/cellular activity was determined by ORAC (AoxC) and in the Caco-2 cell line (CAA), while antidiabetic potential by α-amylase inhibition. The supplementation with processed chickpeas (TG/TGE) increased protein, ash, and isoflavone content (*p* < 0.05) compared with TC. SGD (%) released (*p* < 0.05) bioactive compounds from tortillas, and their bioaccessibility was among 34–70%; noticeably low phenolic bioaccessibility in TG/TGE. The AoxC was higher in TG/TGE (*p* < 0.05) compared with TC; in contrast, CAA was higher in TC, and both increased after SGD. TG depicted the lowest amylase inhibition; after SGD, the IC_50_ values were 62–72-fold lower in the digests than in the tortillas. These results suggest that functional tortillas with processed chickpeas enhance nutraceutical potential.

## 1. Introduction

The increasing prevalence of non-communicable chronic diseases represents a serious problem in healthcare systems, motivating the development of foods that provide health benefits and basic nutrition [1,2]. These are known as functional foods, contain bioactive compounds with additional health effects [3]. Functional foods are like conventional foods, so they are consumed in usual diets [1]. In Mexico, corn tortillas are a good vehicle as functional food for bioactive supplementation, since tortillas are important part of the daily diet. The functional tortillas are usually developed to improve the protein quality by reducing amino acids deficiencies or increasing phytochemical content. These tortillas are supplemented with legumes or other seeds [4,5].

Tortillas are made from maize, and in addition, the formulation of tortilla with pigmented maize may be attracting interest due to nutritional and nutraceutical properties, which may be enhance the potential health benefits by the content of secondary metabolites as the anthocyanins [6,7]. The traditional way to make tortillas is by nixtamalization [4]; however, this process produces changes in the nutritional content, concentration of phytochemicals, antioxidant activity, and the in vitro bioaccessibility of bioactive compounds [8]. An alternative process to reduce these disadvantages of nixtamalization is extrusion, which is a high temperature short time process (HTST) that can reduce the anti-nutritional factors increasing the nutritional value [9,10]. Furthermore, alkaline extrusion is an alternative process that generates masa/flour to develop nixtamalized traditional tortillas, while retaining higher levels of anthocyanins, which may be relevant in blue corn products [11]. Additionally, alkaline extrusion processing offers ecological advantages, as it does not generate nejayote, contrasting with the traditional process, which produces a by-product rich in phytochemicals [11,12].

Chickpea (*Cicer arietinum* L.) is the third most widely produced legume, and it is a good source of protein and contains several bioactive compounds (fiber, flavonoids, and others) with health benefits [13]. There are two chickpea types (kabuli and desi) that exhibit substantial differences in color, chemical composition, size, and geographical distribution [13]. In recent years, the desi-pigmented chickpea has been adapted to the soil conditions in Sinaloa, Mexico. However, this legume remains unexploited despite its potential as a significant source of phytochemicals and good protein quality [14,15].

Food processing influences the release, bioaccessibility, and biological activity of functional foods. Chickpea germination increased the phenolic and isoflavone content with antioxidant potential [15,16]. Moreover, the combination of extrusion and germination processes has been used in rice, sorghum, and chia to improve the microbiological stability, bioactive compound content, and potential health effects [17,18]. However, there are only studies on the release of peptides or phenolic compound in germinated or extruded seed, with limited information on the combination of germination-extrusion or the effect of SGD [16,19].

In addition, the antioxidant properties of chickpea and corn tortilla have been well documented [11,16]; however, the understanding of their biological function in vivo requires evaluating not only their concentration but also their bioaccessibility. The bioavailability measurements in vivo are complex and expensive, so the in vitro digestion models simulating gastrointestinal conditions have become a valuable tool for accessing bioaccessibility as the proportion of a nutrient release from the food matrix and could be available for absorption. The in vitro models offer advantages of reproducibility, simplicity, rapidity, and cost-effectiveness compared to in vivo studies. Also, a strong correlation has been reported in the bioavailability results [20,21].

There are some studies on the fate of bioactive compounds in digestion in corn tortillas, supplemented with legumes or other seeds, or as tacos [4,22,23,24]. But the information still scarcely details all employed different digestion protocols and this makes it difficult to compare results. Nevertheless, despite the development of functional tortillas supplemented with legumes, the effect of processing combination (germination–extrusion) or alkaline extrusion in the in vitro biaccessibility of bioactive compounds continue to be poorly comprehended, in particular for underutilized desi chickpea. Hence, this study aimed to characterize the chemical composition, color, and nutraceutical potential of functional blue corn tortillas supplemented with germinated or germinated–extruded desi chickpea. Moreover, the effect of simulated gastrointestinal digestion on the bioaccessibility of bioactive compounds with potential antioxidant activity, and α-amylase inhibition was determined.

## 2. Materials and Methods

### 2.1. Materials

Chickpea cultivar (green, ICC5613) was grown at the experimental station of the National Research Institute for Forestry, Agriculture and Livestock (INIFAP) in Culiacan, Sinaloa, Mexico. The chickpeas were harvested, cleaned, and stored at −20 °C until processing and analysis. The blue corn was obtained from commercial market in Sinaloa, Mexico.

### 2.2. Lime Cooking Extrusion

The (lime cooked) extruded blue corn flours were achieved following the reported conditions to obtain flours to produce appropriate tortillas [11]. Briefly, corn batches (0.5 Kg) were placed in a blender at low speed to break the kernels. The corn fragments (1–2 mm) were mixed with calcium hydroxide (0.21 g Ca(OH)_2_/100 g) and conditioned at 28% moisture. Each batch was stored at 4 °C/12 h in a polyethylene bag. Extrusion was performed in single screw extruder model 20 DN (Brabernder Instruments Inc., South Hackensack, NJ, USA) with a screw diameter of 19 mm, a length to diameter ratio of 20.1, a nominal compression ratio of 2:1, and a die opening of 2.4 mm. The extruder conditions were 85 °C of extrusion temperature and 240 rpm of screw speed. The extrudates were collected in perforated aluminum trays and cooled and dried at room temperature for 24 h. After that, the samples were milled until able to pass through an 80-mesh sieve. The flours were stored in polyethylene bags at 4 °C until analysis.

### 2.3. Chickpea Germination

The germination process was achieved as previously described [15]. Briefly, chickpea seeds (100 g) were disinfected in 200 mL of 0.12% sodium hypochlorite solution for 3 min and washed 3 times with distilled water. Subsequently, the seeds were hydrated in 0.85 volumes (mL in 100 g) of distilled water for 6 h at 25 °C and shaken every 15 min. Subsequently, the seeds were transferred and dispersed evenly in plastic trays. The germination process was carried out in an incubator at 24 ± 1 °C with a relative humidity of 80% and in darkness. Samples were collected after 5 germination days. The resulting germinated seeds were dried in a dehydrator at 60 °C for 25 h, ground, and stored at −20 °C until analysis.

### 2.4. Chickpea Extrusion

The germinated–extruded chickpea flours were obtained as previously described [25]. Batches of 200 g germinated chickpea flour were conditioned at 26.5% moisture. Extrusion was executed in a single-screw extruder model 20 DN (Brabender Instruments Inc., South Hackensack, NJ, USA). The extrusion conditions were 130 °C of extrusion temperature and 240 rpm of screw speed. The extrudates were collected in perforated aluminum trays, cooled, and dried at room temperature for 24 h. The samples were milled and stored in polyethylene bags at 4 °C until analysis.

### 2.5. Tortilla Preparation

Tortillas were prepared following the methodology previously described [11]. Three functional tortillas were made: TC: alkaline extruded blue corn (100%) tortilla. TG: alkaline extruded blue corn (80%) with germinated desi chickpea (20%) tortilla. TGE: alkaline extruded blue corn (80%) with germinated–extruded desi chickpea (20%) tortilla. The corresponding flour or mixture (200 g) was integrated with water to achieve an appropriate consistency for tortilla production. Masa (30 g per tortilla) was shaped into flat discs using a manual tortilla press. The masa disc was cooked on hot griddle at 290 °C for 27 s each side until the tortillas puffed. The tortillas were cooled at room temperature, dried in a dehydrator at 60 °C for 25 h, ground, and stored at 4 °C until analysis.

### 2.6. Physicochemical Characterization

#### 2.6.1. Proximal Composition

The chemical composition was determinate according to AOAC standard method for moisture (925.09 B), ash (923.03), lipids (954.02), and protein (960.52;N × 6.25) [26]. Total starch (AOAC 996.11) was determined using Megazyme kit K-STA (Wicklow, Ireland).

#### 2.6.2. Color

Color determination was carried out on the tortillas [14], using a Model CR210 colorimeter (Minolta LTD, Japan) with a CIELAB scale, where the following parameters were recorded: L* (0 “dark” − 100 “light”), chromatic coordinates a* (+a*: red, −a*: green), b* (+b*: yellow, −b*: blue), and the values of Chroma C*, hue angle H*, and color differential ΔE will be calculated:

(1) $$C^{*}=\sqrt{\left(a^{*2}+b^{*2}\right)}$$

(2) $$H^{*}={tan}^{-1}\left(\frac{b^{*}}{a^{*}}\right)\times degrees$$

(3) $$\mathrm{if}\;a\;<\;0\;y\;b>0\;H^{*}=180+{tan}^{-1}\left(\frac{b^{*}}{a^{*}}\right)\times degrees$$

(4) $$∆E=\sqrt{{\left(∆L\right)}^{2}+{\left(∆a\right)}^{2}+{\left(∆b\right)}^{2}}$$

### 2.7. Simulated Gastrointestinal Digestion (SGD)

The SGD was carried out according to the method described by Brodkorb et al. (2019) [20] with modifications. Briefly, 5 g of tortilla was dissolved in a 1:1 ratio of water to simulate the bolus, then diluted with simulated salivary fluid (pH 7) with salivary amylase (75 U/mL of digesta). The mixture was incubated at 37 °C and 200 rpm for 2 min, reaching a final volume of 15 mL. The oral bolus was mixed with simulated gastric fluid (pH 3), pepsin (2000 U/mL of digesta) and gastric lipase (60 U/mL of digesta), then incubated at 37 °C with agitation (200 rpm) for 120 min, reaching a final digesta volume of 30 mL. Subsequently, the chyme was mixed with simulated intestinal juice (pH 7), pancreatin (100 U/mL of digesta), and bile (70 mg/g sample); and incubated at 37 °C with agitation (200 rpm) for 120 min, reaching a final digesta volume of 50 mL. Digestion was terminated by heating at 100 °C for 5 min. The digests were centrifugated (13,000× *g* at 4 °C for 15 min) to separate soluble (supernatant) and insoluble (pellet) digests. Finally, digests were freeze dried and stored at −20 °C.

#### Bioaccessibility and Recovery Indices

The Bioaccessibility and recovery indices were calculated as described by Ortega et al. (2011) [27]. The bioaccessibility index (BI) is calculated to estimate the bioactive compounds available for absorption during the digestion process in the human body.

(5) $$\%BI=\left(\frac{SDF}{SDF+IDF}\right)\times 100$$

The recovery index (RI) evaluates the efficiency of bioactive compounds can be extracted or recovered from the SGD.

(6) $$\%RI=\left(\frac{SDF+IDF}{ND}\right)\times 100$$

SDF is the soluble digested fraction, IDF is the insoluble digested fraction, and ND is the non-digested fraction.

### 2.8. Bioactive Compounds Determination

#### 2.8.1. Bioactive Compounds Extraction

Extracts were prepared following the methodology described by Milán-Noris et al. (2018) [15] with some modifications. A total of 50 mg of sample was suspended in 1 mL of monobasic phosphate solution at pH 8, sonicated for 10 min at room temperature, and centrifuged at 4 °C, 3000× *g* for 10 min. The supernatant was collected, and the same procedure was repeated with the remaining pellet. The supernatant was then freeze dried and stored at −20 °C. The peptide fraction from soluble and insoluble digests were fractionated by ultrafiltration using a 10 kDa cut-off hydrophilic membrane (Millipore, Darmstadt, Germany). After that, the peptide fractions (<10 kDa) were obtained, freeze dried, and stored at −20 °C until analysis.

#### 2.8.2. Soluble Protein and Peptide Quantification

Soluble protein quantification was performed using the Detergent Compatible Protein Assay (Thermo Fisher Scientific, Grand Island, NY, USA). Bovine serum albumin was used as a standard at a concentration range of 0.1 to 1 mg/mL. Results were expressed as mg/g of lyophilized sample. The Peptide quantification in soluble and insoluble digest fraction (<10 kDa) was performed by the Quantitative Colorimetric Peptide Assay Kit (Thermo Fisher Scientific, Grand Island, NY, USA. P/N 23275) according to the manufacturer’s protocol.

#### 2.8.3. Total Phenolics and Anthocyanins Content

The total phenolic content (TPC) of the samples was determined using the Folin–Ciocalteau method [28]. Absorbance was measured at 740 nm using a spectrophotometer. Quantification was performed using a gallic acid curve and TPC results were expressed in milligrams equivalent to gallic acid per gram of sample (mg GAE/g). The total anthocyanins content in the tortillas and digested samples was determined as previously reported [11]. A total of 0.5 g of sample was mixed with 1 mL of acidified methanol with 1N HCl (85/15; *v*/*v*). The samples were vortexed and centrifuged at 3000 rpm for 10 min at 4 °C. The supernatant was collected. The absorbances were measured at 536 and 700 nm. The anthocyanins content was reported as milligrams equivalent of cyanidin 3-glucoside per 100 g of sample (mg C3G/100 g) and was calculated using the following formula:

(7) $$C=\frac{\left[\left(A\;535\;nm-A\;700\;nm\right)\right]\left[VM\right]\left[PM\right]}{\left[\varepsilon \right]\left[Sample\;weight\right]}$$

C: Total anthocyanins content concentration (mg cyanidin 3-glucoside equivalent/g), ε: Molecular absorptivity of cyanidin 3-glucoside, VM: Total volume of the sample, PM: Molecular weight of cyanidin 3-glucoside.

#### 2.8.4. Isoflavone Quantification

Isoflavones were quantified in tortillas and digests as previously described [15], using high-performance liquid chromatography (HPLC-DAD) with diode array detection (LC 1260; Agilent technologies, Santa Clara, CA, USA). Separation was achieved using an Eclipse XDB C18 column (3 mm × 150 mm, 3.5 μm; Agilent technologies, Santa Clara, CA, USA) with a flow rate of 0.4 mL/min and a column temperature of 30 °C. A volume of 2 μL was injected and detection was recorded at 260 nm. The mobile phase consisted of a 0.1% solution of formic acid in water (solvent A) and acetonitrile (solvent B). The gradient profile was set up as follows: 0–10% solvent B for 8 min, 10–35% solvent B for 8 min, 35–90% solvent B for 10 min, and 90–100% solvent B for 10 min. Isoflavones were quantified using biochanin-A (10–100 μg/mL; R2 = 0.9994) and formononetin (5–100 μg/mL; R2 = 0.9999) standards. The results were expressed as μg/g lyophilized sample.

### 2.9. Antioxidant Determination

#### 2.9.1. ORAC

The antioxidant capacity of the samples was evaluated using oxygen radical absorbance capacity (ORAC) method [29]. The reagents were added in this order: 25 μL of Trolox standard or samples diluted in phosphate-buffered solution (PBS), 150 μL of fluorescein (70 nM/well), and 60 μL of AAPH (2,2′-azobis(2-methylpropionamidine) at 12 mM/well). The plate was incubated for 30 min at 37 °C prior to AAPH addition. The plate was read in a microplate reader (Bio-Tek Instruments, Winooski, VT, USA), at 485 nm (excitation) and 520 nm (emission). Absorbance was recorded every 2 min until 1 h. Values were expressed as micromoles of Trolox equivalents (TE)/100 g.

#### 2.9.2. Cellular Antioxidant Activity

The determination of cellular antioxidant activity (CAA) was achieved as previously reported [30]. The caco-2 cells (human colorectal adenocarcinoma, HTB-37) were obtained from the American Type Culture Collection (ATCC, Manassas, VA, USA). The caco-2 cells were seeded in a 96-well black plate (100 μL, 5 × 10^4^ cells/well) in Dulbecco’s Modified Eagle’s Medium (DMEM) supplemented with 5% fetal bovine serum and 1% penicillin-streptomycin antibiotic. The tortillas and digests were tested at 50 μg/mL. After 24 h of incubation (37 °C, 5% CO_2_), the cells were washed with PBS and incubated for 20 min with 100 μL of sample and 60 μM of dichlorodihydro-fluorescein diacetate (DCFH-DA) (1:1 *v*/*v*). Then, 100 μL of 500 μM AAPH solution was added to induce oxidative stress. Fluorescence was measuremed at 528 nm emission and 485 nm excitation every 2 min for 45 min at 37 °C. The experiments were performed in three independent trials with three replicates per trial. Cells incubated with DCFH-DA (without sample) and induced with AAPH represent the positive control. CAA was calculated as a percentage using the following equation:

(8) $$\%\mathrm{CAA}\;=\;100-\left[\left(\frac{sample}{positive\;control}\right)\;\times 100\right]$$

### 2.10. Enzyme Inhibition

The α-amylase inhibition assays followed the method previously reported by Vilcacundo et al. (2017) [31]. Briefly, 50 μL of sample, positive control (acarbose) or negative control (distilled water), was mixed with 100 μL of an α-amylase solution (2 U/mL in 0.02 M sodium phosphate buffer pH 6.9). Samples were incubated in a thermomixer C (Eppendorf, Germany) for 5 min. Then, 100 μL of 1% of potato starch solution (dissolved in 0.02 M sodium phosphate buffer at pH 6.9 and boiled for 15 min) was added to each tube and incubated in the thermomixer at 1000 rpm for 6 min. Finally, 100 μL of dinitrosalicylic acid solution was added and tubes were placed in boiling water for 15 min. The 800 μL of distilled water was added and absorbance was read at 540 nm in a microplate reader (Bio-Tek Instruments, Winooski, VT, USA). The percentage of inhibition was calculated using negative control as 100% of enzyme activity. IC_50_ values were obtained using sigmoidal dose–response regression curves.

### 2.11. Statistical Analysis

Statistical analysis was performed using JMP 18 software from SAS Institute (Cary, NC, USA). Data were analyzed using one-way ANOVA followed by Tukey’s test to detect differences between samples. All experiments and measurements were performed in triplicate unless otherwise stated and a *p*-value (<0.05) was considered significant.

## 3. Results and Discussion

### 3.1. Proximal Composition

Table 1 showed the nutritional composition of functional corn tortillas. The TC showed a protein content of 9.56%, similar results were previously reported in tortillas with pigmented maize [32]. The supplementation of tortilla with processed chickpea increased protein content in 2.81% (TG) and 3.46% (TGE) compared to TC. Similar results have been reported in tortillas supplemented with beans and chickpeas [33,34]. The modifications in the protein content could be by the higher content of this macronutrient in chickpea (25–28%) that usually increase in germinated samples [16]. Ash content in the tortillas with processed chickpea was 16% higher than TC. The lipid content in functional tortillas was different among the samples, with higher content in TC. Similar lipid content has been reported in previous works with similar pigmented maize [7]. The starch content was lower in 71.4% (TG) and 66.2% (TGE) compared to 75.6% TC. The other carbohydrates content represented from 9.08 to 17.03% of total composition. It was lower in TC vs. TG and TGE. Since the main carbohydrate in tortilla is starch, this may be related to the content of fiber or free sugars. The supplementation with extruded chickpea in corn tortilla increased the dietary fiber content compared to the control [5].

The color of functional tortillas is shown in Table 1. The color parameters ranged from 37.55 to 44.25 (L*), 4.08 to 4.45 (a*), and 1.79 to 6.23 (b*), which caused the chickpea tortillas to a brighter, and meant that they had a tendency to be more red-yellow tones compared to TC. The difference in color (ΔE) was significantly different in chickpea tortillas compared to TC. Also, the tortillas supplemented with processed chickpea had a difference in Croma values (7.22–7.66 vs. 4.47) and hue angle values (34.83–35.52 vs. 66.45) compared to blue maize tortilla (TC). Other researchers has observed changes in the color parameter than can impact the tortilla acceptability [4].

### 3.2. Effect of Simulated Digestion on Bioactive Compounds in Functional Tortillas

#### 3.2.1. Bioactive Compounds Content in Tortillas and Digests

In Table 2 are depicted the soluble protein (SP) and peptides content in functional tortillas and digests. The tortilla supplemented with processed chickpea (undigested) showed (TG 16% and TGE 18%) lower SP values (*p* < 0.05) compared to TC (Table 2). The SP in soluble digest of tortillas ranged from 79.02 (TG) to 91.31 (TC) mg/g and the insoluble digest from 48.52 (TG) to 78.22 (TGE) mg/g. The recovery index (RI) values ranged from 583.85 (TC) to 897.53 (TGE) % in the functional tortillas. The increase in SP after SGD can be related to the protein hydrolysis in the digestion process in which the protein matrix showed breakdown of peptides and free amino acids. Additionally, the peptide content (<10 kDa) was only determinate in the digested tortillas; in the soluble digest, it ranged from 104.8 (TC) to 230.5 (TG) mg/g and in the insoluble digest from 127.2 (TC) to 214 (TGE) mg/g. The peptide content in chickpea-supplemented tortillas was significantly higher compared to TC in soluble and insoluble digest. This can be associated with the higher content of protein in this tortilla (Table 1), and the processing of chickpea as germination and extrusion that can enhance the liberation of peptides after SGD, and as in other investigations has been observed in germinated chickpea and germinated lupin-corn extrudates [16,35].

The total phenolic content (TPC) in the functional tortillas did not show as being statistically significant, suggesting that the supplementation with processed chickpea did not increase TPC values (Table 3). Likewise, in a supplemented tortilla with ayocote and quintonil lower TPC values than the control tortilla were observed [4], and this reduction may be attributed to antagonist interaction between phenolics compounds, as previously reported in tortillas with processed amaranth [36]. Interestingly, the TPC in the functional tortillas (0.96–1 g GAE /100 g) represents the soluble phenolics extracted by sonication [15]. It seems that this methodology may yield higher amounts of TPC compared to previous reports (19.9 to 20.8 mg GAE/100 g) on extruded blue corn tortillas, as free phenolics [11].

The TPC values in the soluble digest of tortillas ranged from 2.63 (TG) to 4.64 (TC) g GAE/100 g and in the insoluble digest from 2.58 (TC) to 4.90 (TG) g GAE/100 g. The RI values in the tortillas were 717 to 785%, which represents the TPC released from tortillas by SGD. The phenolics compounds are known to have interactions with the macronutrients (protein, starch, and fiber) or be linked, so that after SGD these compounds are free, increasing TPC values [37,38]. Menchaca-Armenta et al. (2023), reported that TPC values increased from 33 to 311.4 mg GAE/100 g after intestinal digestion in extruded nixtamalized blue tortilla, indicating the release of those compounds from the food matrix, as observed in the functional tortillas from this study [22].

The combination of food processing and SGD usually increased the liberation of phenolics compounds. In traditional or alkaline-extrusion tortilla making several processing steps make the corn more digestible [22,39]. Among the phenolics compounds reported in corn and tortilla, the ferulic acid is more abundant [11]. After SGD, some researchers have reported ferulic and gallic acids in the tortilla digests [24,39]. In contrast, other studies had also observed that after SGD of tortillas, the TPC values did not increase [4]. The scarce information on tortilla digestion and the divergent in the digestion methodology makes comparing results complicated. But an important parameter in bioactive quantification is the solubility of the molecules and that may be translated into greater release of the molecules after the digestion.

The total anthocyanin content (TAC), in the tortillas and digests, are depicted in Table 3. The anthocyanins are the compounds that caused the blue color in the tortillas. These compounds are found mainly in the pericarp or aleurone layer of corn and a good part can be retained in tortilla [40]. Similarly to the TPC, the TAC values in the functional tortillas were not statistically different and its content was similar (4.48 to 4.78 mg C3G/100 g) as previously reported: 2.1 to 15.5 mg C3G/100 g in tortillas from blue nixtamalized maize [40].

The anthocyanins are usually found in conjugated (acylated) in corn or tortillas [6,40]. The combination of food processing and SGD may help to release these compounds [37]. The soluble digest of tortillas ranged from 4.49 (TG) to 6.23 (TC) mg C3G/100 g, and in the insoluble digest from 4.34 (TG) to 7.83 (TGE) mg C3G/100 g. The RI values in the tortillas were 184.9 (TG) to 280.94% (TGE), indicating the TAC released after SGD from tortillas compared to undigested tortilla. In this study, TC was obtained by corn alkaline extrusion, and its combination with SGD enhances the solubility of TAC, increasing 43% in the soluble digest compared to its undigested counterpart. In contrast, Menchaca-Armenta et al. (2023) [22] observed a decrease in the anthocyanin content through digestion phases (oral, gastric, and intestinal) with a low recovery in extruded nixtamalized blue tortilla. Its seems that the pH changes during digestion affected the anthocyanin recovery. Correspondingly, the anthocyanins content in cooked black rice indicated that its content is reduced in the intestinal soluble digest compared to undigested rice by the same SGD method [41].

Isoflavones are the main phenolics in chickpea with positive health effects such as the prevention of cancer, obesity, cardiovascular diseases, and diabetes [42]. In previous studies, the content of isoflavones in chickpea significantly increases by the germination process [15]. The two isoflavones detected in the tortillas and digests were biochanin-A and formononetin in their aglycone form, although, isoflavones were not detected in TC (Table 4).

The content of formononetin (49.03 to 110.3 mg/Kg) was higher than biochanin-A (40.08 to 100.31 mg/Kg) in all samples. The total isoflavones was higher in TG in the undigested (198.24 vs. 169.53 mg/Kg) and insoluble digest (200.40 vs. 89.12 mg/Kg) compared to TGE. But the isoflavones content was higher in the soluble digest in TGE compared to TG. In contrast with the other bioactive compounds in this study, only the soluble digest in TGE was higher than its undigested counterpart. Although the RI values were 131% (TG) and 176.8% (TGE), these results indicate that the food processing combination in TGE, specifically germination and extrusion cooking in chickpea, following the SGD released isoflavones from the food matrix but not higher than the undigested tortilla as the other bioactive compounds.

Finally, it is noteworthy to address that the thermal inactivation step may influence the quantifiable amount of thermolabile compounds such as TPC, anthocyanins, and isoflavones. However, the tortillas had already undergone thermal processing, and the applied treatment was considered appropriate to ensure complete enzyme inactivation.

#### 3.2.2. In Vitro Bioaccessibility of Bioactive Compounds in Functional Tortillas

**Figure 1:**
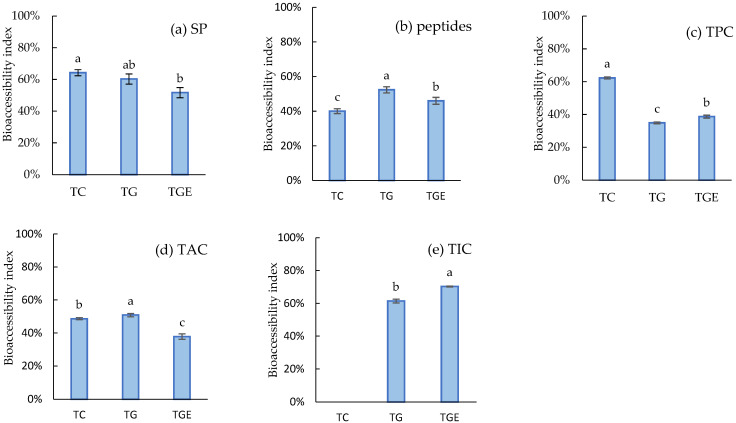
Bioaccessibility index of Soluble protein (SP), peptides, total phenolics (TPC), anthocyanin (TAC), and isoflavones content (TIC) from functional tortillas after simulated gastrointestinal digestion. TC: alkaline extruded blue corn (100%) tortilla. TG: alkaline extruded blue corn (80%) with germinated desi chickpea (20%) tortilla. TGE: alkaline extruded blue corn (80%) with germinated-extruded desi chickpea (20%) tortilla. The results are means ± standard deviation. Means with different letters are statistically different (*p* < 0.05).

The bioaccessibility index (BI) values estimated in this study (Figure 1) were calculated to determinate the percentage of bioactive compounds that may potentially intestinal absorbed in the intestine [27]. Nevertheless, in other studies, BI is usually calculated as percentages of bioactive compounds released from its previous digestion step [22,41]. In addition to the calculation approach and the different SGD protocols, it makes it difficult to compare results between studies. The BI values of soluble protein (Figure 1a) from tortillas were 51.6% (TGE) to 64.2% (TC). While the BI values of peptides content (Figure 1b) from tortillas were 40% (TC) to 52.3% (TG). Interestingly, in TC the SP BI values are the highest and in peptides are the lowest. From previous studies, SP is usually related to the peptide’s formation. In this case, the peptides (<10 kDa) in TC seem to be less absorbable that the chickpea tortillas. The TGE showed low BI values in SP and peptides, which indicates the potential of these compounds to be available at a colonic level. In a previous study, a good amount of anti-inflammatory peptides were found in the insoluble digest of chickpea protein concentrate [15]. Kang et al. (2023) [43] observed a microbiota modulation by chickpea protein and peptides, also reporting a potential antioxidant effect of insoluble proteins.

The IB value of TPC (Figure 1c) in TC (64%) was higher (*p* < 0.05) than tortillas with processed chickpea 34% (TG) and 38% (TGE), which indicates that a good amount of TPC in those tortillas may not be absorbable. Menchaca-Armenta et al. (2023) [22] observed a similar pattern after SGD of extruded blue corn tortilla but with higher BI values. TG and TGE may have prebiotic potential and modulate microbiota as reported in chickpea and tortillas with beans [23,43,44]. Moreover, the BI values of TAC (Figure 1d) from tortillas were 37.8% (TGE) to 50.8% (TG). The BI was lower in TGE (37%), which indicates that a 63% of total digested sample could reach the colon. Some researchers have found that bound phenolics are released by the human colonic microbiota in blue corn tortillas [7] and chickpea [44], and among the compound’s studies are TPC, anthocyanins, and some flavanones such as hesperetin. Also reported is a potential antioxidant effect that may improve colon health.

Isoflavone bioaccessibility (Figure 1e) in the tortillas ranged from 61.4% (TG) to 70.2 (TGE), and it seems that isoflavones in TGE are more bioaccessible which may be because of the combination of process (germination and extrusion). Previous studies have shown that pure isoflavones, such as formononetin have very low BI (1%). Moreover, in the same study, daidzin showed the highest BI in the soy ingredient (68%) and as pure standard (77%) compared to other isoflavones (genistin, daidzein, and ganistein). Additionally, glycoside isoflavones exhibited higher bioaccessibility than aglycone isoflavones [45].

Also, 39% of isoflavones in TG could be metabolized in the colon. Previous studies reported non-absorbable isoflavones, with potential anti-inflammatory activity, from germinated desi chickpea [15]. Chen et al. (2022) [46] reported that soy isoflavones can modulate the gut microbiota and regulate certain groups of probiotic bacteria.

Although the microbiota-related effects were not evaluated in this study, the results suggest that a high level of bioactive compounds of tortillas supplemented with chickpea may not be absorbed in the intestine and could reach the colon. This may indicate a prebiotic potential as previously reported in similar bioactive compounds in chickpea ingredients.

### 3.3. Nutraceutical Potential of Functional Tortillas and Its Digest

Table 5 shows the antioxidant capacity (AoxC) in the functional tortillas and its digest. The tortillas supplemented with processed chickpea showed an increase of 100% (TG) and 71% (TGE) in the antioxidant capacity compared to TC (*p* < 0.05). The increase in AoxC may be due to the supplementation of isoflavones from the processed chickpea since we did not find difference in the other bioactive compounds in the undigested tortilla. Moreover, the AoxC values increased 6 to 13-fold in the soluble digest and 4 to 8.2-fold in the insoluble digest compared to its undigested counterparts. However, no significant differences in AoxC were observed in the soluble and insoluble digest. The AoxC of the tortilla and its digests showed positive correlation with its bioactives compounds, particularly with SP (r = 0.9554, *p* = 0.0001), peptides (r = 0.7756, *p* = 0.0140), and TPC (r = 0.7037, *p* = 0.0344). Several authors have associated an increase in antioxidant capacity with the release of bioactive compounds from the food matrix through the digestion process [22,24]. The AoxC in insoluble digest has been reported in tortilla and chickpea protein, in which the presence of anthocyanins, isoflavones, and peptides showed not only a prebiotic potential but antioxidant that may contribute to colon health [7,43,44].

Furthermore, the cellular antioxidant activity (CAA) was determinate in human Caco-2 cell line (Table 5). The CAA ranged from 29.5 (TGE) to 33.7% (TC) in undigested tortillas. TC showed the highest CAA values compared with tortillas with chickpea, in contrast with the observed in AoxC. In the soluble digest, the CAA values ranged from 82 (TG) to 87% (TC) and in insoluble digest from 85 (TGE) to 89% (TG). Similar to AoxC, the digests showed higher CAA than undigested tortillas. Likewise, the CAA presents a positive correlation with the following bioactive compounds: SP (r = 0.8498, *p* = 0.0037), peptides (r = 0.8914, *p* = 0.0012), and TPC (r = 0.8675, *p* = 0.0024). Other researchers reported the CAA in HT29 cell lines (human colorectal adenocarcinoma) after SGD from germinated chickpea, this effect was also related to peptides and flavonoids [16]. Although, most of the reported antioxidant reported in unabsorbed digest (tortilla or chickpea) are chemical (ORAC, DPPH or others) [7,43,44]. Peptides and isoflavones from insoluble digest from germinated chickpea protein with anti-inflammatory effect have been reported [15].

Additionally, the % amylase enzyme inhibition is shown in Table 5. The potential of the functional tortillas to inhibit 50% of amylase activity varied from 50 to 54 mg/mL, in which TC showed the highest value. Other authors have reported lower IC_50_ values in blue corn tortillas made from diverse nixtamalization process (commercial, nixtamalization, and alkaline extruded) supplemented with amaranth or chickpea [5,36]. After SGD, the IC_50_ of soluble digests ranged from 0.7 to 0.8 mg/mL, corresponding to a 62–72-fold reduction in the amount of sample needed to inhibit enzyme activity, indicating that with a higher bioactive compound content a lower dose is need to inhibit the enzyme activity. The IC_50_ values among TC and TGE did not show significant differences. The IC_50_ amylase values showed a negative correlation with the bioactive compounds, SP (r = −0.8736, *p* = 0.0001), peptides (r = −0.9091, *p* = 0.0007), and TPC (r = −0.8390, *p* = 0.0047). Other researchers had reported that peptides and phenolics compounds in the intestinal digest of a germinated lupin-corn extrudates presented enzyme inhibitory effect to α-amylase and dipeptidyl peptidase IV (DPP-IV). Moreover, the peptides in white and blue tortilla with chickpea hydrolysate showed a potential DPP-IV before and after SGD [47].

The limitations of this study are the in vitro design, which does not reproduce physiological conditions, particularly intestinal absorption, metabolism, and microbiota modulation. Though the results provide useful information on the release of bioactive compounds during SGD, it is relevant to evaluate their relevance in physiological conditions. Future research should focus on in vivo validation and microbiota studies to understand the potential effects of tortilla compounds.

## 4. Conclusions

The blue corn tortilla supplementation with processed chickpea increased protein, ash, isoflavones content and antioxidant capacity. Moreover, the simulated gastrointestinal digestion released bioactive compounds with bioaccessibility of 34% to 70%. A considerable fraction of insoluble compounds could exhibit prebiotic potential. These results suggest that the supplementation of corn blue tortillas with processed chickpeas could be a promising strategy to increase bioactive compounds content with nutraceutical potential, thereby enhancing the health benefits of these tortillas. However, further research on the in vivo bioavailability of these compounds is needed to understand their physiological importance.

## Figures and Tables

**Table 1:** Chemical composition and color in functional tortillas.

	Functional Tortillas		
	TC	TG	TGE
Protein (%) ^1^	9.56 ± 0.22 ^b^	12.37 ± 0.58 ^a^	13.02 ± 0.51 ^a^
Lipid (%) ^1^	3.98 ± 0.17 ^a^	2.76 ± 0.1 ^b^	1.61 ± 0.07 ^c^
Starch (%) ^1^	75.69± 0.61 ^a^	71.43± 0.47 ^b^	66.28± 0.12 ^c^
Ash (%) ^1^	1.66 ± 0.12 ^b^	1.97 ± 0.13 ^a^	2.03 ± 0.04 ^a^
Other Carbohydrates (%) ^1^	9.08± 0.61 ^c^	11.45± 0.93 ^b^	17.03± 0.52 ^a^
Color			
L*	37.55 ± 0.23 ^c^	44.25 ± 0.78 ^a^	41.54 ± 0.96 ^b^
a*	4.08 ± 0.12 ^b^	4.45 ± 0.19 ^a^	4.12 ± 0.02 ^b^
b*	1.79 ± 0.45 ^b^	6.23 ± 0.22 ^a^	5.93 ± 0.41 ^a^
ΔE	0.40 ± 0.16 ^c^	8.06 ± 0.76 ^a^	5.77 ± 0.92 ^b^
C	4.47 ±0.28 ^b^	7.66 ±0.28 ^a^	7.22 ± 0.41 ^a^
H	66.45 ± 4.74 ^a^	35.52± 0.44 ^b^	34.84 ± 2.22 ^b^

^1^ dry basis. TC: alkaline extruded blue corn (100%) tortilla. TG: alkaline extruded blue corn (80%) with germinated desi chickpea (20%) tortilla. TGE: alkaline extruded blue corn (80%) with germinated-extruded desi chickpea (20%) tortilla. The results are the means ± standard deviation. Means with different letters in the same row are statistically different (*p* < 0.05).

**Table 2:** Soluble protein (SP) and peptides in functional tortillas before and after simulated gastrointestinal digestion.

	Functional Tortillas		
	TC	TG	TGE
SP (mg/g)			
Tortilla	24.46 ± 1.18 ^a^	20.33 ± 0.55 ^b^	19.92 ± 1.13 ^b^
Soluble digest	91.31 ± 1.25 ^a^	79.02 ± 6.38 ^b^	82.64 ± 4.52 ^b^
Insoluble digest	51.5 ± 3.06 ^b^	48.82 ± 3.16 ^b^	78.22 ± 4.17 ^a^
Total digest	142.81 ± 2.52 ^a^	127.84 ± 3.79 ^b^	160.87 ± 18.62 ^a^
RI (%)	583.85 ± 33.49 ^b^	628.82 ± 29.33 ^b^	897.53 ± 87.10 ^a^
Peptides (<10 kDa) (mg/g)			
Tortilla	N.d.	N.d.	N.d.
Soluble digest	104.8 ± 3.0 ^c^	230.5 ± 17.9 ^a^	182.9 ± 9.3 ^b^
Insoluble digest	127.2 ± 9.1 ^b^	209.6 ± 10.8 ^a^	214.8 ± 7.0 ^a^
Total digest	226.0 ± 10.30 ^c^	440.1 ± 24.2 ^a^	397.7 ± 5.1 ^b^

N.d. Not determined. TC: alkaline extruded blue corn (100%) tortilla. TG: alkaline extruded blue corn (80%) with germinated desi chickpea (20%) tortilla. TGE: alkaline extruded blue corn (80%) with germinated-extruded desi chickpea (20%) tortilla. RI. Recovery index. The results are means ± standard deviation. Means with different letters in the same row are statistically different (*p* < 0.05).

**Table 3:** Total phenolics (TPC) and anthocyanin (TAC) content in functional tortillas before and after simulated gastrointestinal digestion.

	Functional Tortillas		
	TC	TG	TGE
TPC (g GAE/100 g)			
Tortilla	1.00 ± 0.02 ^a^	0.96 ± 0.04 ^a^	0.96 ± 0.05 ^a^
Soluble digest	4.64± 0.17 ^a^	2.63 ± 0.03 ^b^	2.90 ± 0.12 ^c^
Insoluble digest	2.58 ± 0.07 ^c^	4.90 ± 0.04 ^a^	4.60 ± 0.08 ^b^
Total digest	7.41 ± 0.16 ^a^	7.48 ± 0.06 ^a^	7.34 ± 0.04 ^a^
RI (%)	717.67 ± 33.28 ^a^	785.33 ± 39.78 ^a^	779.56 ± 64.34 ^a^
TAC (mgC3G/100 g)			
Tortilla	4.64 ± 0.04 ^a^	4.78 ± 0.1 ^a^	4.48 ± 0.08 ^a^
Soluble digest	6.23 ± 0.53 ^a^	4.49 ± 0.0 ^b^	4.77 ± 0.31 ^b^
Insoluble digest	6.58 ± 0.21 ^a^	4.34 ± 0.17 ^b^	7.83 ± 0.17 ^c^
Total digest	12.76 ± 0.26 ^a^	8.84 ± 0.17 ^b^	12.6 ± 0.34 ^a^
RI (%)	275.9 ± 4.17 ^a^	184.90 ± 6.66 ^b^	280.94 ± 3.00 ^a^

TC: alkaline extruded blue corn (100%) tortilla. TG: alkaline extruded blue corn (80%) with germinated desi chickpea (20%) tortilla. TGE: alkaline extruded blue corn (80%) with germinated-extruded desi chickpea (20%) tortilla. RI. Recovery index. The results are means ± standard deviation. Means with different letters in the same row are statistically different (*p* < 0.05).

**Table 4:** Isoflavones content in functional tortillas before and after simulated gastrointestinal digestion.

	Functional Tortillas		
	TC	TG	TGE
Isoflavones (mg/Kg)			
Formononetin			
Tortilla	ND	100.74 ± 1.13 ^a^	97.54 ± 0.03 ^b^
Soluble digest	ND	84.24 ± 2.71 ^b^	110.32 ± 0.06 ^a^
Insoluble digest	ND	54.11 ± 2.95 ^a^	49.03 ± 0.20 ^b^
Total digest		138.35 ± 5.65 ^b^	159.36 ± 0.26 ^a^
Biochanin-A			
Tortilla	ND	97.54 ± 0.03 ^a^	72.00 ± 0.99 ^b^
Soluble digest	ND	75.33 ± 1.28 ^b^	100.31 ± 0.0 ^a^
Insoluble digest	ND	46.29 ± 4.49 ^a^	40.08 ± 0.90 ^a^
Total digest		121.62 ± 5.76 ^b^	140.39 ± 0.89 ^a^
Total Isoflavones			
Tortilla	ND	198.24 ± 1.10 ^a^	169. 54 ± 0.95 ^b^
Soluble digest	ND	159 ± 3.98 ^b^	210.63 ± 0.06 ^a^
Insoluble digest	ND	100.40 ± 7.44 ^a^	89.12 ± 1.10 ^b^
Total digest		259.97 ± 11.42 ^b^	299.75 ± 1.04 ^a^
RI (%)		131.13 ± 6.48 ^a^	176.80 ± 1.6 ^b^

ND. Not detected. TC: alkaline extruded blue corn (100%) tortilla. TG: alkaline extruded blue corn (80%) with germinated desi chickpea (20%) tortilla. TGE: alkaline extruded blue corn (80%) with germinated-extruded desi chickpea (20%) tortilla. RI. Recovery index. The results are means ± standard deviation. Means with different letters in the same row are statistically different (*p* < 0.05).

**Table 5:** Antioxidant and anti-diabetic potential of functional tortillas before and after simulated gastrointestinal digestion.

	Functional Tortillas		
	TC	TG	TGE
Antioxidant capacity (ORAC, mmol TE/100 g)			
Tortilla	2.69 ± 0.11 ^b^	5.40 ± 0.57 ^a^	4.63 ± 0.35 ^a^
Soluble digest	37.37 ± 1.08 ^a^	34.25 ± 1.37 ^a^	33.60 ± 1.96 ^a^
Insoluble digest	22.16 ± 1.95 ^a^	21.23 ± 0.68 ^a^	22.14 ± 1.24 ^a^
Cellular Antioxidant Activity (%) ^1^			
Tortilla	33.73 ± 1.53 ^a^	31.03 ± 1.30 ^b^	29.51 ± 1.49 ^b^
Soluble digest	87.92 ± 3.25 ^a^	82.06 ± 1.63 ^b^	83.43 ± 1.50 ^ab^
Insoluble digest	87.86 ± 0.68 ^ab^	89.98 ± 0.66 ^a^	85.97 ± 2.35 ^b^
α-amylase inhibition (IC_50_: mg/mL)			
Tortilla	55.35 ± 1.47 ^b^	50.27 ± 1.43 ^a^	52.79 ± 1.87 ^ab^
Soluble digest	0.72 ± 0.01 ^a^	0.81 ± 0.01 ^b^	0.69 ± 0.02 ^a^

^1^ Caco-2 (sample dose: 50 µg/mL. TC: alkaline extruded blue corn (100%) tortilla. TG: alkaline extruded blue corn (80%) with germinated desi chickpea (20%) tortilla. TGE: alkaline extruded blue corn (80%) with germinated-extruded desi chickpea (20%) tortilla. The results are means ± standard deviation. Means with different letters in the same row are statistically different (*p* < 0.05).

## Data Availability

The raw data supporting the conclusions of this article will be made available by the authors upon request.
